# Is Copper-61 the New Gallium-68? Automation and Preclinical Proof-of-Concept of ^61^Cu-Based Radiopharmaceuticals for Prostate Cancer Imaging

**DOI:** 10.3390/ph18040469

**Published:** 2025-03-26

**Authors:** Diana Rodrigues, Alexandra I. Fonseca, Sérgio do Carmo, José Sereno, Ivanna Hrynchak, João N. Moreira, Célia Gomes, Antero Abrunhosa

**Affiliations:** 1 Coimbra Institute for Biomedical Imaging and Translational Research, and Institute for Nuclear Sciences Applied to Health (CIBIT/ICNAS), University of Coimbra, 3000-548 Coimbra, Portugal; dianarodrigues@icnas.uc.pt (D.R.); sergiocarmo@uc.pt (S.d.C.); josesereno@uc.pt (J.S.); 2 ICNAS Pharma, University of Coimbra, 3000-548 Coimbra, Portugal; alexandrafonseca@icnas.uc.pt (A.I.F.); ivanna.ua@icnas.uc.pt (I.H.); 3 Center for Neuroscience and Cell Biology (CNC), University of Coimbra, 3004-504 Coimbra, Portugal; jmoreira@ff.uc.pt; 4 Faculty of Pharmacy, University of Coimbra, 3000-548 Coimbra, Portugal; 5 Centre for Innovative Biomedicine and Biotechnology Consortium (CIBB), University of Coimbra, 3000-548 Coimbra, Portugal; cgomes@fmed.uc.pt; 6 Faculty of Medicine, Coimbra Institute for Clinical and Biomedical Research (iCBR), University of Coimbra, 3000-548 Coimbra, Portugal; 7 Clinical Academic Center of Coimbra (CACC), 3000-075 Coimbra, Portugal

**Keywords:** copper-61, radiopharmaceutical, automation, positron emission tomography, prostate cancer, prostate specific membrane antigen

## Abstract

**Background:** While gallium-68 has traditionally dominated PET imaging in oncology, copper radionuclides have sparked interest for their potential applications in nuclear medicine and theranostics. Considering the advantageous physical decay properties of copper-61 compared to those of gallium-68, we describe a fully automated GMP-compliant synthesis process for ^61^Cu-based radiopharmaceuticals and demonstrate their in vivo application for targeting the overexpressed PSMA by PET/MR imaging. **Methods:** Copper-61 was obtained through the irradiation of natural zinc liquid targets in a biomedical cyclotron. [^61^Cu]Cu-DOTAGA-PSMA-I&T and [^61^Cu]Cu-NODAGA-PSMA-I&T were produced without manual intervention in two Synthera^®^ Extension modules. Radiochemical purity was analyzed by radio-HPLC and iTLC. Cellular uptake was evaluated in LNCaP and DU145 cells. In vivo PET/MRI was performed in control mice to evaluate the biodistribution of both radiopharmaceuticals, and in tumor-bearing mice to assess the targeting ability towards PSMA. **Results:** The fully automated process developed proved to be effective for the synthesis of ^61^Cu-based radiopharmaceuticals, with appropriate molar activities. The final products exhibited high radiochemical purity (>98%) and remained stable for up to 6 h after the EOS. A time-dependent increase in cellular uptake was observed in LNCaP cells, but not in DU145 cells. As opposed to [^61^Cu]Cu-NODAGA-PSMA-I&T, [^61^Cu]Cu-DOTAGA-PSMA-I&T exhibited poor kinetic stability in vivo. Subsequent PET/MR imaging with [^61^Cu]Cu-NODAGA-PSMA-I&T showed tumor uptake lasting up to 4 h post-injection, predominant renal clearance, and no detectable accumulation in non-targeted organs. **Conclusions:** These results demonstrate the feasibility of the implemented process, which yields adequate amounts of high-quality radiopharmaceuticals and can be adapted to any standard production facility. This streamlined approach enhances reproducibility and scalability, bringing copper-61 closer to widespread clinical use, to the detriment of the conventionally accepted gallium-68.

## 1. Introduction

Prostate cancer (PCa) is the second most commonly diagnosed malignancy and ranks as the fifth leading cause of cancer-related deaths amongst men worldwide [1], thus representing a major burden in terms of both morbidity and mortality rates [2]. The development of accurate tools for early detection and effective surveillance is essential to improve patient outcomes.

In fact, significant progress has been made since the approval by the U.S. Food and Drug Administration (FDA) of the first monoclonal antibody (mAb)-based, prostate specific membrane antigen (PSMA)-targeted radioligand—ProstaScint^®^ (Indium-111 Capromab Pendetide)—for SPECT imaging of PCa [3,4,5,6,7,8], back in 1996. Building on the positive results, subsequent efforts have focused on developing PET tracers to leverage this superior imaging modality. In addition, mAb-based radiopharmaceuticals have limited access to tumors and remain in circulation in the human body for prolonged periods of time [9]. Therefore, smaller molecules with more favorable pharmacokinetic profiles are preferred. These considerations have led to extensive research and subsequent evaluation of a vast number of peptide-based PSMA inhibitors as well as different positron-emitting radionuclides [10,11,12,13,14,15,16]. Recently, two PSMA-targeted radiopharmaceuticals for PET imaging of PCa received FDA approval: [^68^Ga]Ga-PSMA-11 in December 2020 [17] and [^18^F]DCFPyL in May 2021 [18]. These radiopharmaceuticals have since become the most widely used for imaging PSMA-expressing PCa in the clinical setting.

Regardless of its favorable physical decay characteristics, i.e., high positron branching ratio and low positron energies, which per se produce PET images with improved spatial resolution, fluorine-18 (t_1/2_ = 110 min; 97% β^+^; 3% EC; E_max β+_ = 0.634 MeV) has a rather complex chemistry and the radiolabeling of the molecules of interest is not straightforward [19], which ultimately hinders the development and validation of fluorine-based radiopharmaceuticals in a reasonable time frame. On the other hand, metal radionuclides, e.g., gallium-68 (t_1/2_ = 68 min; 89% β^+^; 11% EC; E_max β+_ = 1.899 MeV) [20], have a simple and fast coordination chemistry [21], easily adjustable to any type of vector (e.g., antibodies and their fragments, peptides, nanoparticles, etc.) [14,22,23,24,25,26,27,28]. The availability of ^68^Ge/^68^Ga generators, granting the supply of gallium-68 without the need for an on-site cyclotron, have prompted the interest in ^68^Ga-conjugated radiopharmaceuticals [21] and accelerated their translation from the bench to the bedside. However, generators are expensive and their production under good manufacturing practice (GMP) conditions is limited [29,30]. Furthermore, the low activity yield per elution, along with a 3–4 h dead time between elutions, restrain large scale productions [20,31], making it insufficient to meet the global growing demand. As the limitations of ^68^Ge/^68^Ga generators outweighed their advantages over alternative production routes, there has been a significant shift towards exploring other metal radionuclides beyond gallium-68 [15,16,22,23,32,33,34], even though its production at a cyclotron is also feasible and able to produce significantly higher activities [35,36].

Copper-61 (t_1/2_ = 3.33 h; 61% β^+^; 39% EC; E_max β+_ = 1.216 MeV) [20] is an emerging radionuclide with highly favorable characteristics for PET imaging, easily produced in a cost-effective manner through the irradiation of natural zinc liquid targets [37]. Moreover, copper-61 offers several advantages over gallium-68, including (1) lower positron emission energies, (2) a longer half-life that matches the biological half-life of small molecules and facilitates the distribution of radiopharmaceuticals to distant research and clinical centers without on-site cyclotrons, and (3) its pairing with the therapeutic counterpart copper-67, essential as we gradually step into the era of theranostics [20]. These attributes make copper-61 a promising candidate for the next generation of PET radiopharmaceuticals, which will most likely benefit from the replacement of gallium-68 by this copper radionuclide. Indeed, significant efforts have been devoted to the development and validation of new ^61^Cu-based radiopharmaceuticals [14,28], highlighting its potential and relevance in this field.

Herein, we provide a detailed description of a fully automated synthesis method to produce ^61^Cu-labeled PSMA-targeted radiopharmaceuticals designed for routine clinical applications. Implementing automated synthesis methodologies for radiopharmaceutical development is essential to ensure precision, efficiency, and reproducibility, while minimizing radiation exposure to operators. In addition, we present a preclinical evaluation of [^61^Cu]Cu-NODAGA-PSMA-I&T as a PET imaging tool in a mouse model of PCa. Through this work, we offer valuable insights into a highly effective and streamlined manufacturing process, while advocating for the use of copper-61 as a reliable alternative to the gold standard gallium-68 in the near future.

## 2. Results

### 2.1. Automated Radiopharmaceutical Synthesis and Radiochemical Purity

Irradiation of natural zinc liquid targets with the conditions described in the Section 4.1 yielded between 350–500 MBq of purified [^61^Cu]CuCl_2_, which was directly used in the radiolabeling reactions. PSMA-based radiopharmaceuticals were obtained with radiochemical yields (RCY; decay-corrected) of 90.4 ± 1.6% and molar activities of 17.7 ± 2.6 MBq/nmol (N = 3). More details are presented in supplemental material—Table S1.

**Figure 1:**
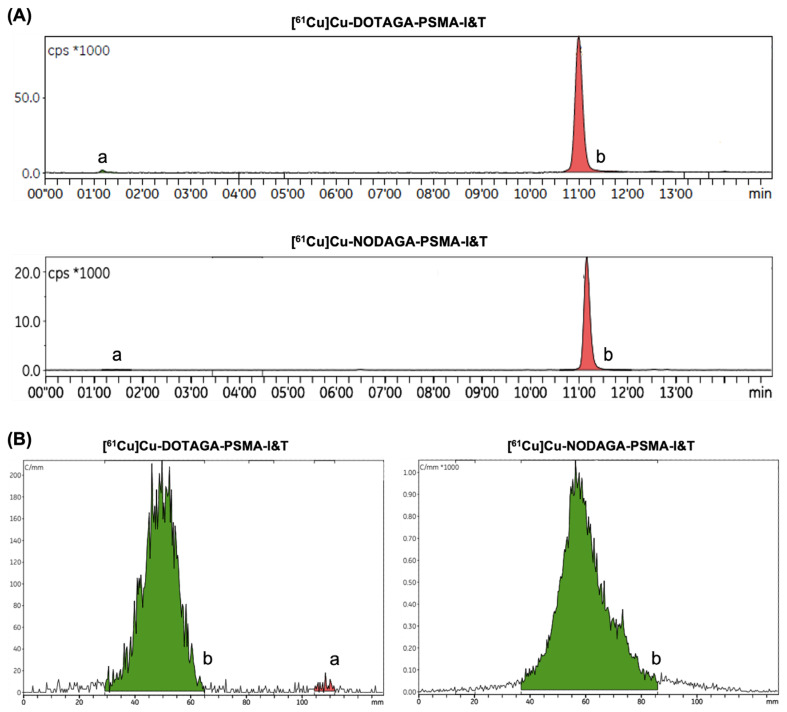
Representative (**A**) radio-HPLC and (**B**) iTLC chromatograms attesting the radiochemical purity of the final radiopharmaceuticals for [^61^Cu]Cu-DOTAGA-PSMA-I&T (upper row; left column) and [^61^Cu]Cu-NODAGA-PSMA-I&T (lower row; right column), in which “a” represents free copper-61 and “b” represents the radiolabeled compounds.

The radiochemical purity (evaluated by radio high-performance liquid chromatography (HPLC) and instant thin layer chromatography (iTLC)) was confirmed to be above 98% at the end of synthesis (EOS) for both radiopharmaceuticals, as shown in Figure 1. Slight differences in the retention factor of the radiolabeled compounds in the iTLC chromatograms are likely due to minor variations in the elution length of the strips. No radiolysis was observed when working within this range of activities.

### 2.2. In Vitro Stability and Uptake Studies

**Figure 2:**
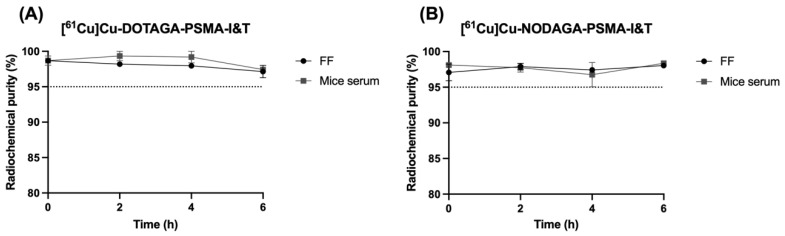
Stability results for (**A**) [^61^Cu]Cu-DOTAGA-PSMA-I&T and (**B**) [^61^Cu]Cu-NODAGA-PSMA-I&T in the final formulation and after incubation with mice serum over a period of 6 h after the EOS. Data are expressed as mean ± SEM (N = 3–6). FF: final formulation.

The stability of the PSMA-based radiopharmaceuticals in the final formulation and mice serum was evaluated up to 6 h after the EOS. Both [^61^Cu]Cu-DOTAGA-PSMA-I&T and [^61^Cu]Cu-NODAGA-PSMA-I&T demonstrated excellent stability, maintaining radiochemical purities above 95% throughout the considered period, as depicted in Figure 2.

**Figure 3:**
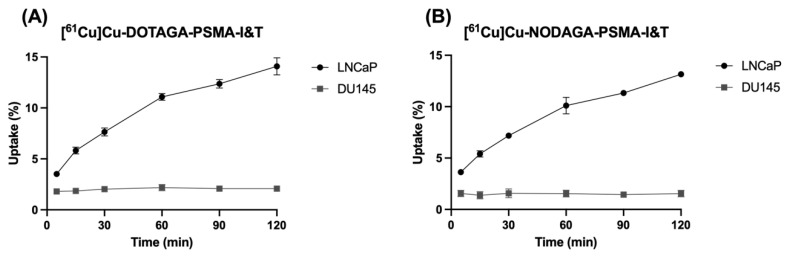
Cellular uptake of (**A**) [^61^Cu]Cu-DOTAGA-PSMA-I&T and (**B**) [^61^Cu]Cu-NODAGA-PSMA-I&T in PSMA-positive (LNCaP) and PSMA-negative (DU145) cells. Data are presented as mean ± SEM (N = 2–5).

To evaluate the specific uptake of [^61^Cu]Cu-DOTAGA-PSMA-I&T and [^61^Cu]Cu-NODAGA-PSMA-I&T via PSMA, LNCaP (PSMA-positive) and DU145 (PSMA-negative) cells were incubated with 0.5 nM of either radiopharmaceutical. Cellular uptake was measured at different time points up to 2 h. In LNCaP cells, uptake of both [^61^Cu]Cu-DOTAGA-PSMA-I&T and [^61^Cu]Cu-NODAGA-PSMA-I&T showed a time-dependent increase, reaching maximum levels of 14.09 ± 0.84% and 13.16 ± 0.15%, respectively, at the later time points. Conversely, uptake in DU145 cells remained at basal levels throughout the whole experiment, as shown in Figure 3.

### 2.3. PET/MR Imaging in Healthy Mice

**Figure 4:**
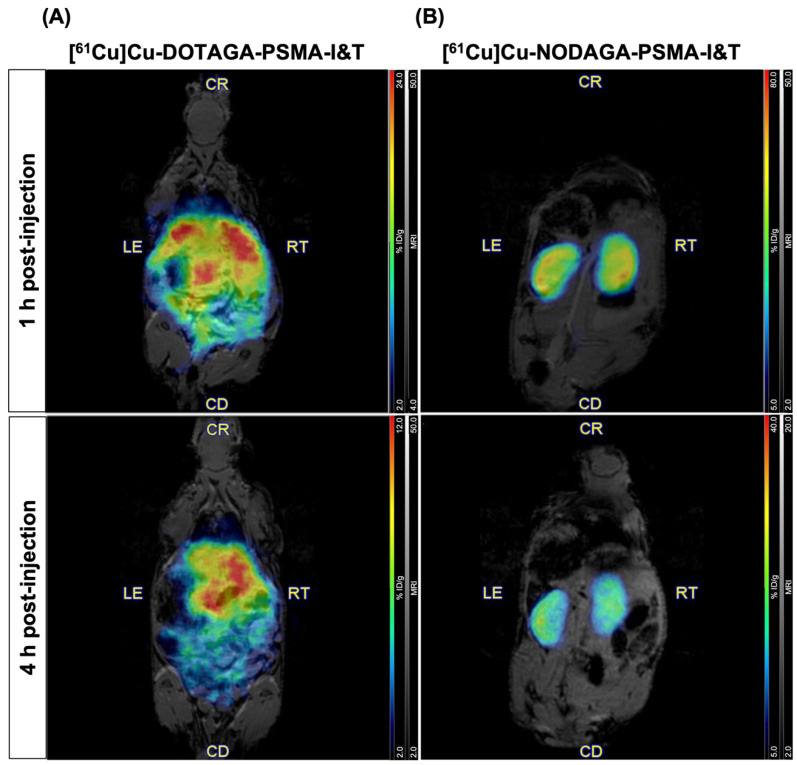
Representative coronal whole-body PET/MR images acquired at 1 h (upper row) and 4 h (lower row) post-injection of (**A**) [^61^Cu]Cu-DOTAGA-PSMA-I&T (N = 2) and (**B**) [^61^Cu]Cu-NODAGA-PSMA-I&T (N = 1) into the tail vein of control mice. Images were normalized to % ID/g.

The in vivo biodistribution of both [^61^Cu]Cu-DOTAGA-PSMA-I&T and [^61^Cu]Cu-NODAGA-PSMA-I&T was evaluated in healthy control mice by PET/MRI. Whole-body images were acquired at 1 h and 4 h post intravenous injection of the radiopharmaceuticals. The biodistribution profiles of the two radiolabeled compounds differed significantly, as shown in Figure 4. [^61^Cu]Cu-DOTAGA-PSMA-I&T was predominantly retained in the abdominal region, particularly in the liver and gastrointestinal tract (Figure 4A), whereas [^61^Cu]Cu-NODAGA-PSMA-I&T showed a more favorable pattern of uptake, with prominent accumulation in the kidneys (Figure 4B) and the urinary bladder. These results were validated by ex vivo biodistribution studies performed immediately after image acquisition at the latest time point. While [^61^Cu]Cu-NODAGA-PSMA-I&T was confirmed to accumulate preferably in the kidneys and urine with residual uptake in non-targeted organs, [^61^Cu]Cu-DOTAGA-PSMA-I&T exhibited a very scattered accumulation in various non-targeted organs, including the liver, stomach, and small and large intestines (Figure S1). The biodistribution profile of [^61^Cu]Cu-DOTAGA-PSMA-I&T resemble that of [^61^Cu]CuCl_2_, consistent with excretion via the hepatobiliary system, and previously reported by Bernabeu et al. [14]. These findings suggest that the stability of [^61^Cu]Cu-DOTAGA-PSMA-I&T may be compromised in vivo, as copper-61 appears to undergo transchelation and decomplexation from the DOTAGA chelator. Considering these results, PET/MR imaging studies in tumor-bearing mice were conducted with [^61^Cu]Cu-NODAGA-PSMA-I&T.

### 2.4. Imaging of PSMA Receptors with [^61^Cu]Cu-NODAGA-PSMA-I&T

**Figure 5:**
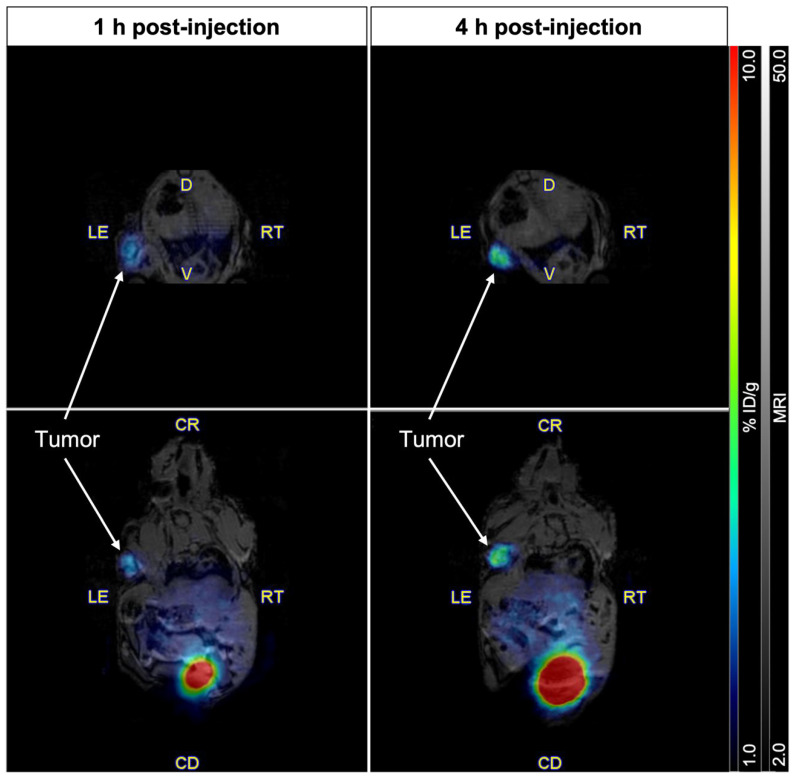
Representative axial (upper row) and coronal (lower row) whole-body PET/MR images acquired at 1 h (left column) and 4 h (right column) post-injection of [^61^Cu]Cu-NODAGA-PSMA-I&T into the tail vein of LNCaP tumor-bearing mice. Images were normalized to % ID/g.

PET/MR images acquired 1 h and 4 h after intravenous injection of [^61^Cu]Cu-NODAGA-PSMA-I&T into the tail vein of mice bearing PSMA-positive tumor xenografts are shown in Figure 5. At 1 h post-injection, a selective accumulation of the radiopharmaceutical was clearly visible in the left shoulder, co-localizing precisely with the PSMA-expressing tumor mass visualized by MRI. At 4 h post-injection, a marked increase in signal intensity was observed and supported by PET quantification, which revealed a significant rise in tumor uptake from 3.27% to 6.00% ID/g over this time period. Consistent with the observations in control mice, no radiopharmaceutical accumulation was detected in non-targeted organs or tissues, apart from the kidneys (not pictured) and the urinary bladder. Uptake in the kidneys, urine, and tumors was further confirmed by ex vivo biodistribution (Figure S1).

## 3. Discussion

Given the exponential increase in the demand for metal-based radiopharmaceuticals for PET imaging over the last decade, this study addresses two major challenges within the fields of radiopharmacy and radiopharmaceutical development: the need for potential alternatives to conventional ^68^Ga-labeled PET tracers that could provide improved efficiency and cost-effectiveness, and the development of fully automated methods for the routine production thereof.

In this context, the use of copper-61 in nuclear medicine offers several advantages, as its physical decay properties appear to be more appropriated than those of the quintessential PET radionuclide gallium-68. Copper-61 has a moderately higher half-life (3.33 h vs. 68 min), offering an optimal compromise between PET imaging protocol requirements and production/distribution feasibility. Furthermore, it emits positrons with lower energies which result in less scattering and attenuation of the emitted radiation, leading to improved imaging quality. Not only do these intrinsic attributes of copper-61 facilitate the supplying of radiopharmaceuticals over larger geographic areas, but they also meet the requirements of a forthcoming generation of PET tomographs with increased spatial resolution. Lastly, it is important to highlight that the availability of copper-67 [38,39], a therapeutic counterpart to copper-61, further justifies the attention it has garnered in recent years. Although copper-67 is currently scarce, growing interest in this isotope is expected to prompt an increase in the demand and ultimately lead to its regular production in more facilities. This is particularly relevant as we transition into an era of theranostic applications and personalized medicine, where both diagnostics and therapeutics are tailored to individual patients.

The production of copper-61 from natural zinc liquid targets may offer greater convenience compared to the use of solid targets. In addition to avoiding the need for specialized, extremely expensive equipment and dedicated facilities, this approach further minimizes the time-consuming and rather complex post-processing of the irradiated target material. Moreover, natural zinc is also tremendously cheaper than the enriched zinc-64 liquid targets, making it an appealing option for the early preclinical stages of radiopharmaceutical development. The reduced cost allows researchers to focus on addressing practical automation-related issues as well as optimizing the radiolabeling conditions to produce ^61^Cu-based radiopharmaceuticals in an affordable manner. However, the use of natural zinc liquid targets does have its drawbacks. The most significant challenge pertains to scalability. In the present study, we detail the production of approximately 400 MBq of ^61^Cu-based radiopharmaceuticals, which represents a relatively low amount of activity for reliably assessing radiolysis and limits their application to about three patient doses (according to Bernabeu et al. [14]) or fewer, depending on the geographic area to which the radiopharmaceuticals would be distributed to. This issue could potentially be mitigated by adjusting the irradiation parameters (e.g., increasing the time of irradiation and/or the beam current) or by using enriched liquid targets. As previously reported by our group, irradiating enriched zinc-64 liquid targets results in the production of twice the amount of copper-61 compared to using natural zinc liquid targets [28,37]. The yield is even significantly higher if solid targets—either natural or enriched—are used [14,40,41]. Furthermore, since the abundance of zinc-64 in natural zinc is limited to roughly 48.6% (which explains the lower production yields), some copper impurities are inevitably produced alongside copper-61 during irradiation [37]. These impurities arise from nuclear reactions involving zinc-67 and zinc-68, both present in natural zinc at 4.1% and 18.8% [37], respectively, and cannot be separated from the purified [^61^Cu]CuCl_2_ solution. Specifically, around 2.2% of copper-64 is present as a byproduct at the end of bombardment (EOB) when irradiating natural zinc liquid targets [37], whereas nearly no copper impurities are expected to be co-produced when using enriched zinc-64 liquid targets. This phenomenon directly impacts the radionuclidic purity and, consequently, the shelf life of the final radiopharmaceutical, as the copper-64 to copper-61 ratio gradually increases over time [28,37]. All these considerations must be weighed carefully when selecting the most suitable production route, acknowledging the trade-offs involved.

Considering the extensive research on novel radionuclides with the potential to outperform gallium-68, as well as the advantages of producing copper-61 from natural zinc liquid targets allied to the urgent need of improving the prognosis of PCa patients, we have focused on the production of [^61^Cu]Cu-PSMA-I&T. We presented a fully automated synthesis process to produce ^61^Cu-based PSMA-targeted PET imaging probes, comprising both the purification of copper-61 and the radiolabeling of the peptide-based precursors DOTAGA-PSMA-I&T and NODAGA-PSMA-I&T. The entire procedure, including the post-processing of the irradiated solution and subsequent radiopharmaceutical synthesis, was accomplished by using two Synthera^®^ Extension modules and disposable cassettes with appropriate silicone tubing systems, without manual intervention. This approach prevents the risk of radioactive cross-contamination throughout the whole procedure. Additionally, automation ensures high levels of process reliability and consistency, reducing the potential for disruptions in the routine production of radiopharmaceuticals while minimizing the hazards associated with exposure to ionizing radiation [42]. Given the results obtained, the implemented automated process has proven to be feasible, with highly reproducible outcomes, and is fully compliant with GMP standards, a key factor for the validation and clinical translation of radiopharmaceuticals. Not least, this automated method can be easily adapted to other commercially available modules and is suitable for facilities equipped with different setups.

Both radiolabeled compounds exhibited high stability in vitro, along with strong binding and selectivity for PSMA, as shown by the in vitro uptake studies in PCa cells. Uptake increased progressively over time in PSMA-expressing cells, while remaining minimal in PSMA-negative counterparts. On the other hand, [^61^Cu]Cu-NODAGA-PSMA-I&T demonstrated superior performance in vivo as compared to its analogue [^61^Cu]Cu-DOTAGA-PSMA-I&T. PET/MR imaging studies with [^61^Cu]Cu-NODAGA-PSMA-I&T in control mice showed radiopharmaceutical uptake exclusively in the kidneys and the urinary bladder. Conversely, the release of copper-61 from the DOTAGA chelator led to high accumulation of radioactivity in non-targeted organs, such as the liver and gastrointestinal tract, due to excretion of free-copper through the hepatobiliary system [14,25]. These findings are consistent with a recent report on the same radiopharmaceutical in a similar animal model [14], and with previous studies using copper-64 and the DOTA chelator [43,44,45], whose core structure is nearly identical to that of DOTAGA, except for the glycine group. In comparison, [^61^Cu]Cu-NODAGA-PSMA-I&T exhibited remarkable in vivo metabolic stability with clearance predominantly through the renal pathway, which is the preferred excretion route for the washout of small molecules, minimizing potential off-target toxicity due to non-specific accumulation of the radionuclide in healthy organs. This biological stability highlights the high quality of [^61^Cu]Cu-NODAGA-PSMA-I&T as a radiopharmaceutical. Most importantly, [^61^Cu]Cu-NODAGA-PSMA-I&T proved to be highly effective in detecting small PSMA-positive PCa lesions. The anatomical localization of the tumors obtained by MRI perfectly matched the areas of selective radiopharmaceutical accumulation, as clearly visualized on PET images. The ex vivo biodistribution profiles of both radiopharmaceuticals corroborated the observed findings. Specifically, [^61^Cu]Cu-NODAGA-PSMA-I&T was predominantly present in the kidneys, urine, and tumors, reflecting its targeted uptake. In contrast, [^61^Cu]Cu-DOTAGA-PSMA-I&T exhibited a widespread organ accumulation, further denoting significant differences in the biodistribution patterns between the two compounds. The values obtained for each organ closely align with those reported in the literature [14]. Minor discrepancies may be attributed to variations in the total amount of ligand administered or differences in tumor size. As a final point, it is important to give some consideration to the rapid clearance of the radiopharmaceutical. In the context of theranostic applications, a fast clearance is beneficial for imaging purposes (i.e., diagnosis or monitoring), as it allows for quick and transient detection of lesions while avoiding prolonged exposure to patients. However, it can pose a significant obstacle when aiming for sustained therapeutic efficacy, as the rapid elimination of vectors may prevent them from maintaining the high bioavailability necessary to achieve a strong therapeutic effect. This presents a challenge when considering [^61/67^Cu]Cu-NODAGA-PSMA-I&T for clinical translation. To mitigate this concern, strategies aimed at enhancing the pharmacokinetic properties of small-molecule drug conjugates, as well as optimizing their biodistribution and blood residence time, can be employed. For example, introducing albumin-binding or transthyretin-binding entities into otherwise short-lived radiopharmaceuticals may help improve their therapeutic effectiveness [46,47]. Notwithstanding this hurdle, the present study confirms the usefulness of [^61^Cu]Cu-NODAGA-PSMA-I&T as a PET tracer, and provides valuable insight into the optimal timing for image acquisition. The observed increase in tumor uptake over time suggests that delayed imaging beyond the typical 1 h to 4 h post systemic administration, not feasible with shorter-lived isotopes like gallium-68, might improve diagnostic accuracy. However, further studies are needed to fully characterize the kinetic profile of the radiopharmaceutical and to determine the ideal scan timing, balancing maximal tumor accumulation/retention with effective clearance of the radiolabeled molecules from non-targeted tissues.

## 4. Materials and Methods

To prevent metal contaminations, all chemicals and solvents used for [^61^Cu]CuCl_2_ purification and ligand radiolabeling were trace metal grade [hydrochloric acid (HCl) > 37% and nitric acid (HNO_3_) > 69% (Honeywell Fluka; Charlotte, NC, USA), bi-distilled water (BBraun; Melsungen, Germany), ethanol (EtOH; Rotem; Israel), and ammonium acetate (NH_4_OAc; Honeywell Fluka)]. Solvents used for radio-HPLC runs were HPLC grade.

Zinc nitrate hexahydrate (Puratronic™, 99.998%) was purchased from Alfa Aesar (Haverhill, MA, USA). Cassettes and disposable tubing kits used for purification and radiolabeling were acquired from Fluidomica (Cantanhede, Portugal). Purification cartridges including the CU resin and the strong anion exchange (SAX) resin were obtained from Triskem (Bruz, France). Strata-X cartridges were purchased from Phenomenex (Torrance, LA, USA). DOTAGA-PSMA-I&T acetate was purchased from ABX (Radeberg, Germany) and NODAGA-PSMA-I&T acetate was manufactured by Pepmic (Suzhou, China), both fractioned upon arrival and stored at −20 °C in aqueous solution.

### 4.1. [^61^Cu]CuCl_2_ Production and Purification

Copper-61 was produced and purified following the previously described methodology [37,48]. Briefly, 10 mM HNO_3_ was used to dissolve zinc nitrate into a solution with a final zinc concentration of 200 mg/mL, which was then irradiated with a 16.9 MeV proton beam for 60 min in an IBA Cyclone 18/9 medical cyclotron (Louvain-la-Neuve, Belgium). Electrical current was kept between 65−75 μA. At the EOB, the target solution was line-transferred directly to a shielded hot-cell for subsequent purification.

**Figure 6:**
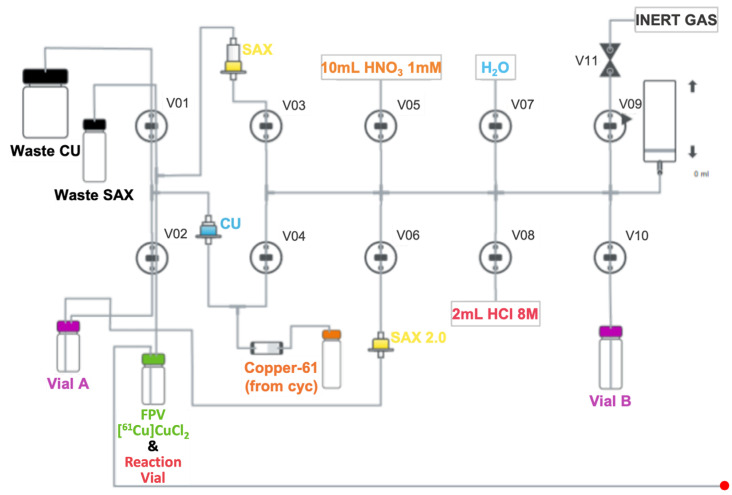
Schematic flow of Synthera^®^ Extension module for post-processing of the irradiated solution. The red dot connects to the red dot in Figure 7. CU: CU cartridge; SAX: strong anion exchange cartridge; FPV: final product vial; cyc: cyclotron; V01–V11: valves 1–11.

The purification of copper-61 was accomplished using a three-step chromatographic, GMP-compliant method (Figure 6) in a Synthera^®^ Extension module (IBA; Louvain-la-Neuve, Belgium) as follows:

- Prior to use, the SAX cartridge is preconditioned with water (10 mL) followed by HCl 8 M (8 mL);
- Water (3mL) is added to the radioactivity-receiver vial to adjust pH;
- The CU cartridge is preconditioned with water (10 mL);
- After the irradiated solution is transferred into the hot-cell, the CU cartridge is loaded using a peristaltic pump to avoid cross-contamination of the tubing system with zinc;
- The CU cartridge is rinsed with HNO_3_ 1 mM (10 mL) and dried for 2 min;
- The CU cartridge is eluted with HCl 8M (2 mL) to Vial A containing 3.3 mL of water;
- The solution is then passed through a smaller SAX cartridge (SAX 2.0—1 mL cartridge; 0.40–0.45 g of resin) in order to remove residual zinc and transferred to Vial B containing > 5.5 mL of HCl 30%;
- Finally, the second SAX cartridge (2 mL cartridge; full) is loaded, dried for 2 min, and eluted with 3 mL of water into the final product vial (FPV).

**Figure 7:**
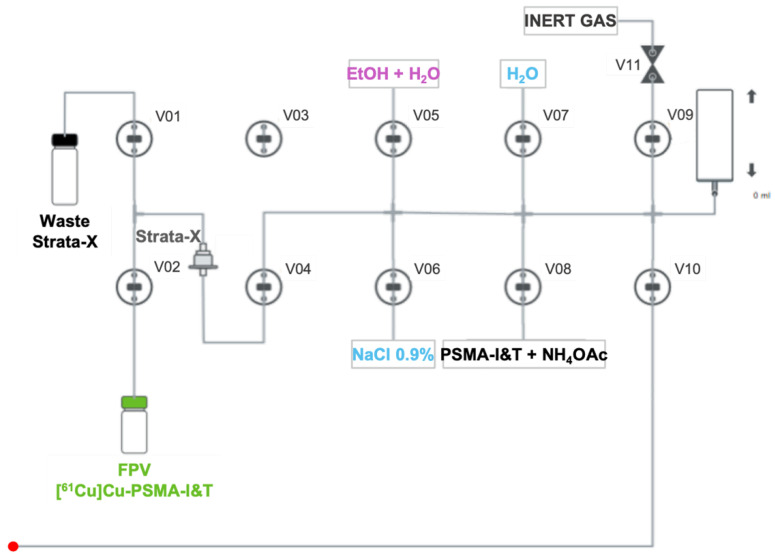
Schematic flow of Synthera^®^ Extension module for the synthesis of [^61^Cu]Cu-PSMA-I&T radiopharmaceuticals. The red dot connects to the red dot in Figure 6. Strata-X: cartridge used for purification; FPV: final product vial; V01–11: valves 1–11.

The process was completed within 35 min from the EOB with RCY of 76.1 ± 4.1% (decay-corrected). After post-processing, the obtained [^61^Cu]CuCl_2_ solution was ready for use in radiopharmaceutical synthesis (Figure 7).

### 4.2. Automated Synthesis of PSMA-Based Radiopharmaceuticals

To optimize the radiolabeling reaction and subsequent purification process prior to automation, different buffers and purification cartridges were tested. Further details can be found in the supplemental material (Figure S2). Temperature, pH, and reaction time were set in accordance with gathered knowledge from previous research within the group. Based on these optimizations, the radiopharmaceutical synthesis process, represented in Figure 7, was completed within 25 min from the end of purification (EOP) using a second Synthera^®^ Extension module (IBA; Louvain-la-Neuve, Belgium) as detailed below:

- A mass of 30 µg of DOTAGA/NODAGA-PSMA-I&T diluted in 5 mL of NH_4_OAc 2 M is transferred to the reaction vial (Figure 6) and mixed with [^61^Cu]CuCl_2_;
- The radiolabeling reaction occurs for 10 min at 100 °C with pH fixed between 3.5- 4.5;
- After that time, the reaction mixture is diluted with water (5 mL) and passed through a Strata-X cartridge into the waste container to remove free copper-61;
- The Strata-X cartridge is rinsed with water (5 mL) and eluted with 2 mL of a water/EtOH solution (1:1) into the FPV;
- Finally, 8 mL of NaCl 0.9% is added to the FPV in order to get the radiopharmaceutical in its injectable final formulation.

### 4.3. Radiochemical Purity

The radiochemical purity was assessed by radio-HPLC and iTLC, not only to ensure the quality of the final radiopharmaceuticals, but also to monitor the efficiency of the automated synthesis process. Details of the equipment and the non-isocratic radio-HPLC method employed are described in Table 1. With regard to the iTLC method, a solution of ethylenediaminetetraacetic acid (EDTA) 0.1 M was used as the mobile phase and glass microfiber chromatography paper strips impregnated with silica gel (iTLC-SG, Agilent; Santa Clara, CA, USA) were used as the stationary phase. Equipment specifications and detailed methodology are also outlined in Table 1.

### 4.4. Stability

The stability of the ^61^Cu-based radiopharmaceuticals was evaluated in the final formulation [EtOH/NaCl 0.9% 1:9 (*v*/*v*)] and in Swiss nude mice serum. For the final formulation, aliquots of [^61^Cu]Cu-DOTAGA-PSMA-I&T and [^61^Cu]Cu-NODAGA-PSMA-I&T were incubated at 37 °C and analyzed by radio-HPLC at specific time points up to 6 h after the EOS. To evaluate the stability in mice serum, 100 μL of [^61^Cu]Cu-DOTAGA-PSMA-I&T or [^61^Cu]Cu-NODAGA-PSMA-I&T was mixed with 400 μL of serum and incubated at 37 °C. At the same time points, 50 μL of the mixture was added to 150 μL of cold EtOH and centrifuged at 3000 rpm, 4 °C, for 10 min to precipitate plasma proteins. The supernatant was then further diluted in NaCl 0.9% and likewise analyzed as stated above.

### 4.5. Cell Culture

The human PCa cell lines LNCaP (PSMA-positive) (American Type Culture Collection (ATCC), CRL-1740; Manassas, VA, USA) and DU145 (PSMA-negative) (kindly provided by Dr. Carmen Jerónimo Lab at IPO-Porto) were maintained at 37 °C in a humidified incubator enriched with 5% carbon dioxide. The cells were grown in Roswell Park Memorial Institute (RPMI) 1640 medium (Sigma-Aldrich, R4130; St. Louis, MI, USA) supplemented with 0.2% (*w*/*v*) sodium bicarbonate (Sigma-Aldrich, S6297), 10% heat-inactivated fetal bovine serum (FBS; Gibco, A5256801; Grand Island, NY, USA), 1 mM sodium pyruvate (Gibco, 11360-070), and 1% antibiotic-antimycotic (Anti-Anti 100X, Gibco, 15240-062).

### 4.6. In Vitro Uptake Assay

LNCaP and DU145 cells were seeded in 12-well plates (0.3 × 10^6^ cells/well/mL) 48 h before the experiments and the day before, respectively. The multiwell plates used for the LNCaP cell line were previously coated with poly-l-lysine (PLL, Innoprot; Bizcaia, Spain) for 4–6 h to prevent cell detachment during the assays. Both PSMA-positive and -negative cells were incubated with 0.5 nM of ^61^Cu-radiopharmaceuticals at 37 °C for different time periods up to 2 h. At each time point, the supernatant was collected, and the cells were washed with phosphate-buffered saline (PBS) and incubated with sodium hydroxide 0.5 M for cell lysis. The radioactivity of both the supernatant and cellular fraction was measured in a CRC-55tW gamma-well counter (Mirion Technologies; Atlanta, GA, USA). Cellular uptake was calculated as a percentage of the total radioactivity measured for each well. The experiments (N = 2–5) were conducted in duplicate.

### 4.7. Experimental Mouse Model of PCa

Animal studies were approved by the Animal Welfare Committee of the Institute of Nuclear Sciences Applied to Health (ICNAS; ORBEA 01-2018) and the Portuguese National Authority for Animal Health (DGAV). Male athymic Swiss nude mice aged 9-12 weeks (34–36 g) were supplied by ICNAS, housed under pathogen-free conditions in individually ventilated cages with controlled temperature/humidity (22 °C/55%) environment on a 12 h light–dark cycle, and provided with food and water ad libitum. Animals (N = 2) were subcutaneously inoculated with LNCaP cells [5 × 10^6^ cells in cold PBS mixed with Corning^®^ Matrigel^®^ Matrix (354262, Corning, NY, USA) at a ratio of 3:1 to a final volume of 150 μL] on the left shoulder. Tumor growth was monitored with a digital caliper and PET/MRI scans were performed when tumor xenografts reached 5–7 mm in diameter (~75–130 mm^3^, as calculated by the formula Length × Width^2^ × 0.5), approximately 9 weeks after inoculation.

### 4.8. In Vivo PET/MR Imaging

[^61^Cu]Cu-DOTAGA-PSMA-I&T (~0.37 MBq/g; ~3.56 nmol; N = 2) or [^61^Cu]Cu-NODAGA-PSMA-I&T (~0.37 MBq/g; ~0.653 nmol; N = 3) in NaCl 0.9% with 10% EtOH was intravenously injected into the tail vein of anesthetized mice. PET images were acquired 1 h and 4 h post-injection in a prototype high-resolution small-animal PET scanner based on resistive plate chambers (RPC-PET) [49], with the animals under anesthesia (1.8–2.0% isoflurane). Respiratory rate and body temperature were monitored throughout the imaging procedures (SA Instruments, Inc.; Stony Brook, NY, USA). Five fiducial markers were placed in the animal bed for optimal PET/MRI co-registration. After PET scanning, a whole-body MRI was performed on a BioSpec 9.4 T MRI scanner (Bruker BioSpin; Ettlingen, Germany) with a body volume resonator (transmitter/receiver, BMRIDE T9361V3/0060), with the animal in the same position for anatomical co-localization. MRI was performed using a multi-slice localizer in coronal orientation with the following parameters (respiratory trigger): TE/TR = 2.008/253.736 ms, field-of-view (FOV) = 80.0 ∗ 45.0 mm, acquisition matrix = 400 ∗ 200, averages = 5, flip angle = 30°, 50 continuous slices with 0.5 mm thickness and acquisition time of 2 min 7 s. The 2D T2-weighted turbo RARE sequence had the following specifications: TE/TR = 22/3000 ms, FOV = 80.0 ∗ 45.0 mm, acquisition matrix = 320 ∗ 320, averages = 3, rare factor = 4, echospacing = 11 ms, 50 coronal continuous slices with 0.5 mm thickness, and acquisition time of 12 min. PET images were reconstructed using the OSEM algorithm and isotropic voxels of 0.5 mm width. For quantitative PET imaging processing, PMOD v3.6 and FUSION Tool (PMOD Technologies; Zürich, Switzerland; RRID:SCR_016547) were used to delineate volumes of interest (VOIs) on the PET images co-registered with the respective MRI. Regions of interest (ROIs) were defined manually based on the anatomical MR images. Uptake values were expressed as percentage of injected dose per gram of tissue (% ID/g).

## 5. Conclusions

The automated synthesis process described proved to be highly efficient and robust, allowing the reproducible production of top-notch ^61^Cu-based radiopharmaceuticals for imaging of PSMA-expressing PCa.

The irradiation of natural zinc liquid targets is a particularly attractive option for copper-61 production as it avoids the use of enriched target material, thus significantly reducing the cost of radiopharmaceutical development, independently of which production route is the most appropriated for future purposes (e.g., in a clinical setting).

With this work, we aim to encourage the use of copper-61 for PET imaging based on its favorable physical decay properties as compared to those of the currently used gallium-68. Such properties enable the generation of images with increased spatial resolution, thereby enhancing scan accuracy and diagnostic precision.

## Figures and Tables

**Table 1:** Radio-HPLC and iTLC methods used to identify and determine radiochemical impurities in the ^61^Cu-based radiopharmaceuticals. Specifications of the HPLC and iTLC equipment, including the model and operational conditions of the method, are provided. Water: HPLC-grade water; TFA: trifluoroacetic acid.

HPLC Equipment Specifications			
**Model**	Agilent 1260 Infinity II		
**Detector**	VWD 1260 Infinity II G7114A		
**Column**	Avantor^®^ ACE^®^ C18, 150 × 3 mm, 3 μm		
**Acquisition software**	Gina X		
**Operating conditions of the system**			
**Wavelength**	264 nm		
**Volume injected**	20 μL		
**Mobile phase A**	Water/TFA 0.1% (*v*/*v*)		
**Mobile phase B**	Acetonitrile/TFA 0.1% (*v*/*v*)		
**Method characterization**	Time (min)	Mobile phase A (%)	Mobile phase B (%)
	0	80	20
	15	70	30
**Running time**	15 min		
**Flow**	0.6 mL/min		
**iTLC equipment specifications**			
**Model**	miniGita		
**Detector**	Scintillation		
**Acquisition software**	TLC Control Software, version 2.30		
**Operating conditions of the system**			
**Chromatographic strips**	iTLC-SG		
**Volume applied**	5 μL		
**Mobile phase**	EDTA 0.1 M		
**Elution length**	9 cm (origin: 1.5 cm from the bottom end; elution front: 1 cm from the top end)		
**Running time**	5 min		

## Data Availability

The datasets that were used and/or analyzed in this study can be found within the article and its Supplementary Materials. For additional information, please make a reasonable request to the corresponding author.

## Supplementary Materials

The following supporting information can be downloaded at: https://www.mdpi.com/article/10.3390/ph18040469/s1. Table S1. Decay-corrected RCY and radiochemical purity of [^61^Cu]Cu-PSMA-I&T radiopharmaceuticals. ^1,2^Assessed by radio-HPLC and iTLC, respectively. RCY: radiochemical yield. Figure S1. Ex vivo biodistribution data of [^61^Cu]Cu-DOTAGA-PSMA-I&T in healthy control mice (dark bars) and [^61^Cu]Cu-NODAGA-PSMA-I&T in LNCaP tumor-bearing mice (light bars) determined by gamma-counting after the 4 h PET/MR imaging time point. Values were normalized to percentage of injected dose per gram of tissue (% ID/g) and expressed as mean ± SEM (N = 2 for each dataset). N.A.: not applicable. Figure S2. Different buffers (A) and purification cartridges (B) tested to optimize the radiolabeling conditions of ^61^Cu-based radiopharmaceuticals and their subsequent purification. The optimal buffer was selected based on the efficiency of the radiolabeling reaction, whereas the purification method was chosen by evaluating the recovery yield, purity, and radiochemical integrity (both not shown) of the final products. Values were expressed as mean ± SEM (N = 3 for each dataset). NaOAc: sodium acetate; NH_4_Ac: ammonium acetate; HLB: hydrophilic/lipophilic balanced.

## Abbreviations

EDTA	Ethylenediaminetetraacetic acid
EOB	End of bombardment
EOS	End of synthesis
EtOH	Ethanol
FDA	Food and Drug Agency
FOV	Field of view
FPV	Final product vial
HCl	Hydrochloric acid
HLB	Hydrophilic/lipophilic balanced
HNO_3_	Nitric acid
HPLC	High-performance liquid chromatography
iTLC	Instant thin layer chromatography
mAb	Monoclonal antibody
MRI	Magnetic resonance imaging
NaOAc	Sodium acetate
NH_4_OAc	Ammonium acetate
PBS	Phosphate-buffered saline
PCa	Prostate cancer
PET	Positron emission tomography
PSMA	Prostate specific membrane antigen
RCY	Radiochemical yield
SAX	Strong anion exchange
SPECT	Single-photon emission computed tomography
