# Supplementary material for: Is Copper-61 the New Gallium-68? Automation and Preclinical Proof-of-Concept of 61Cu-Based Radiopharmaceuticals for Prostate Cancer Imaging
Source: Pharmaceuticals (Basel). 2025 Mar 26;18(4):469. doi: 10.3390/ph18040469 (PMC12030277; doi:10.3390/ph18040469)
Supplement: Supplementary file 1 [file pharmaceuticals-18-00469-s001.zip › pharmaceuticals-3524773-supplementary.pdf]

Supplemental Material

Table S1. Decay-corrected RCY and radiochemical purity of [^{61}Cu]Cu-PSMA-I&T radiopharmaceuticals. [1,2]Assessed by radio-HPLC and iTLC, respectively. RCY: radiochemical yield.

	Activity of [^{61}Cu]$CuCl_2$ (MBq)	Activity of [^{61}Cu]Cu-PSMA-I&T (MBq)	Molar activity (MBq/nmol)	RCY (%)	Radiochemical Purity (%)[1]	Radiochemical Purity (%)[2]
**N = 1**	368.35	339.53	15.79	92.18	98.50	99.08
**N = 2**	400.34	357.12	16.61	89.20	99.27	100.00
**N = 3**	492.10	442.15	20.57	89.85	98.63	98.82

Ex vivo biodistribution studies

Immediately after the 4 h imaging time point, mice were sacrificed by cervical dislocation and perfused with PBS. The tumor (if applicable) and major organs including brain, lungs, heart, liver, spleen, stomach, kidneys, and small and large intestine were harvested. Samples of muscle, bone, blood, urine, and feces were also collected. Each sample was weighted, and its radioactivity measured in a CRC-55tW gamma well counter (Mirion Technologies; Atlanta, GA, USA) and decay-corrected to the time of injection. A calibration curve was obtained to convert units (MBq to cpm or vice versa).

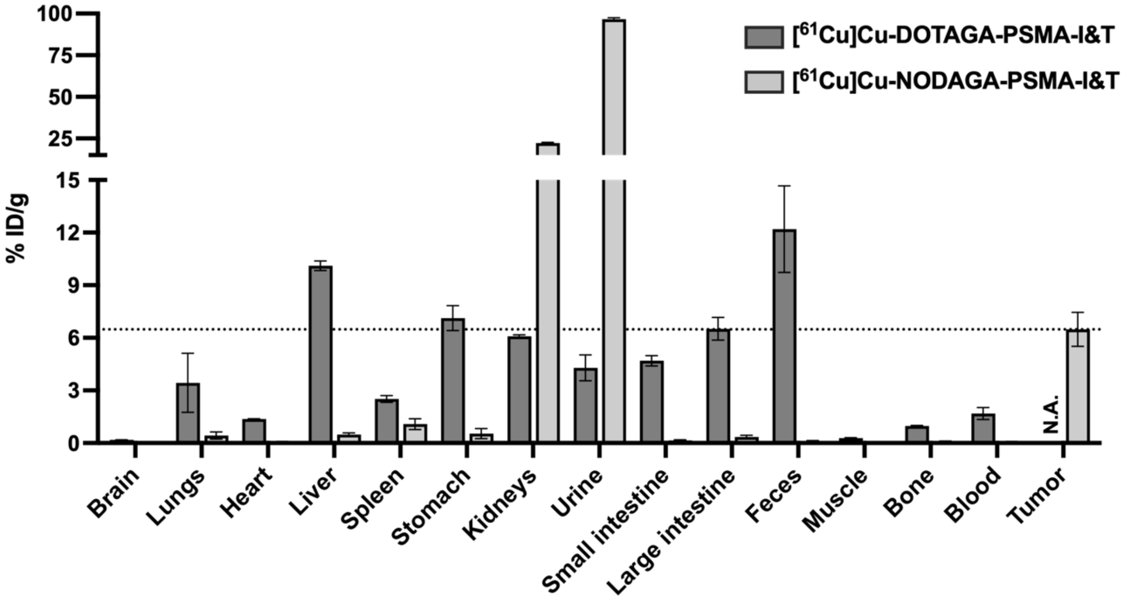

Figure S1. Ex vivo biodistribution data of [^{61}Cu]Cu-DOTAGA-PSMA-I&T in healthy control mice (dark bars) and [^{61}Cu]Cu-NODAGA-PSMA-I&T in LNCaP tumor-bearing mice (light bars) determined by gamma-counting after the 4 h PET/MR imaging time point. Values were normalized to percentage of injected dose per gram of tissue (% ID/g) and expressed as mean ± SEM (N = 2 for each dataset). N.A.: not applicable.

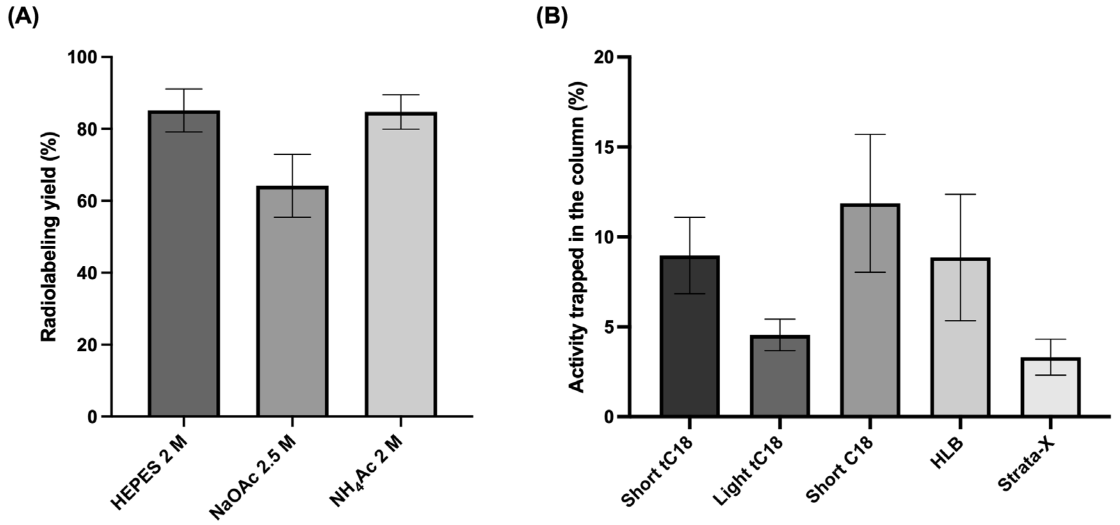

Figure S2. Different buffers (A) and purification cartridges (B) tested to optimize the radiolabeling conditions of ^{61}Cu-based radiopharmaceuticals and their subsequent purification. The optimal buffer was selected based on the efficiency of the radiolabeling reaction, whereas the purification method was chosen by evaluating the recovery yield, purity, and radiochemical integrity (both not shown) of the final products. Values were expressed as mean ± SEM (N = 3 for each dataset). NaOAc: sodium acetate; NH_4Ac: ammonium acetate; HLB: hydrophilic/lipophilic balanced.
